# Lesson of the month: Large vessel vasculitis: A rare cause of transaminitis

**DOI:** 10.1016/j.clinme.2024.100035

**Published:** 2024-03-29

**Authors:** Sumaya Hussein, Greg Allister, Azeem Ahmed

**Affiliations:** Department of Acute medicine, Great Western Hospital, Swindon, United Kingdom

## Abstract

We present the case of a 73-year-old male with pyrexia of unknown origin (PUO). He was a returned traveller from Southern Africa and underwent extensive investigation to rule out an infective cause. This was mostly unrevealing but there was a notable transaminitis (ALT predominant) with normal bilirubin level. He showed no serological or clinical improvement despite antibiotic treatment. Subsequent CT-PET showed high mural uptake in the thoracic and abdominal aorta and its major branches, confirming the diagnosis of Large Vessel Vasculitis (LVV). This case highlights the importance of considering LVV in patients with PUO and with transaminitis.

## 1 Case presentation

A 73-year-old man presented to hospital with a 4-week history of fever, night sweats, weight loss and frontal headache. There was no jaw claudication or visual disturbance. He had recently returned from holiday in Southern Africa. Reported alcohol use was within medically recommended limits. Systems enquiry revealed no other focal symptoms. Clinical examination revealed tenderness over the frontal sinus, but the rest of the cardiac, respiratory, GI and neurological examination was normal.

Inflammatory markers were raised with CRP 159 mg/L (reference range (RR): 0–5 mg/L) and ESR >100 mm in hr (RR: 0–30 mm in 1 h). Two sets of blood cultures showed no growth, as did a urine culture. Procalcitonin result suggested a low risk of bacterial infection. COVID 19 PCR was negative and HIV and EBV serology were negative. TB interferon-gamma release assay was negative, as well as negative thick and thin films for malarial parasites. Liver function tests revealed ALT 364 U/L (RR: 0–50 U/L), ALP 156 U/L (RR: 30–120 U/L) and serum bilirubin within the normal range. Viral hepatitis screen was negative. ANA and ANCA profile were negative. Lactate dehydrogenase level was 619 U/L (RR: 208–378 U/L).

CT head with contrast revealed minor acute pansinusitis only. Abdominal USS showed fatty liver changes only. Contrast-enhanced CT of the thorax, abdomen and pelvis showed nothing to explain his presentation.

At admission broad-spectrum antibiotic therapy was initiated. LFT derangement was seen on admission bloods so antibiotic induced hepatitis was unlikely. Several days into his admission persistent fever and raised inflammatory markers prompted referral to Rheumatology. CT-PET scan was organised, which demonstrated high mural uptake in the thoracic and abdominal aorta and its major branches, particularly the subclavian, carotid and vertebral arteries, confirming the diagnosis of LVV.

**Fig. 1:**
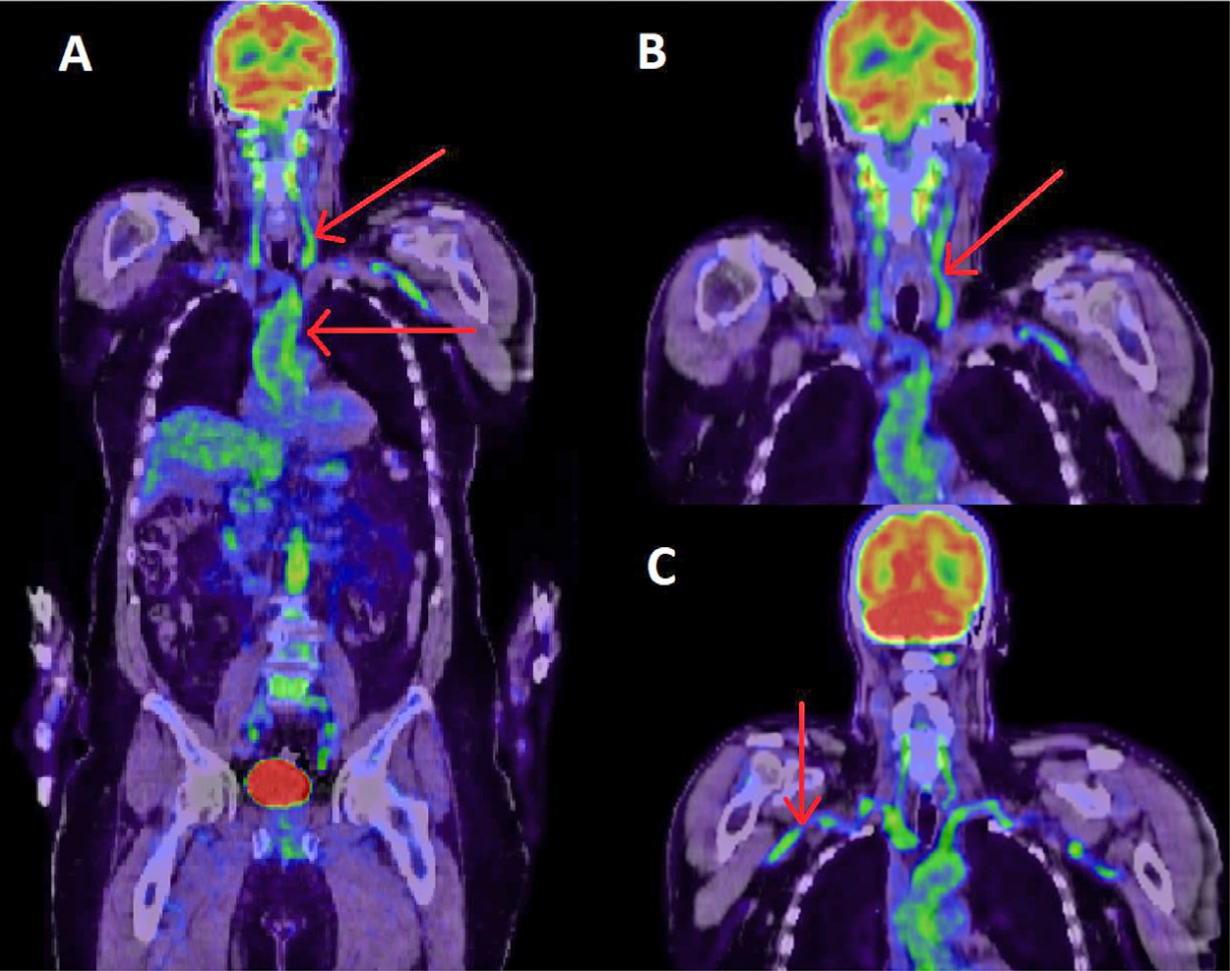
CT-PET results.

**Fig. 2:**
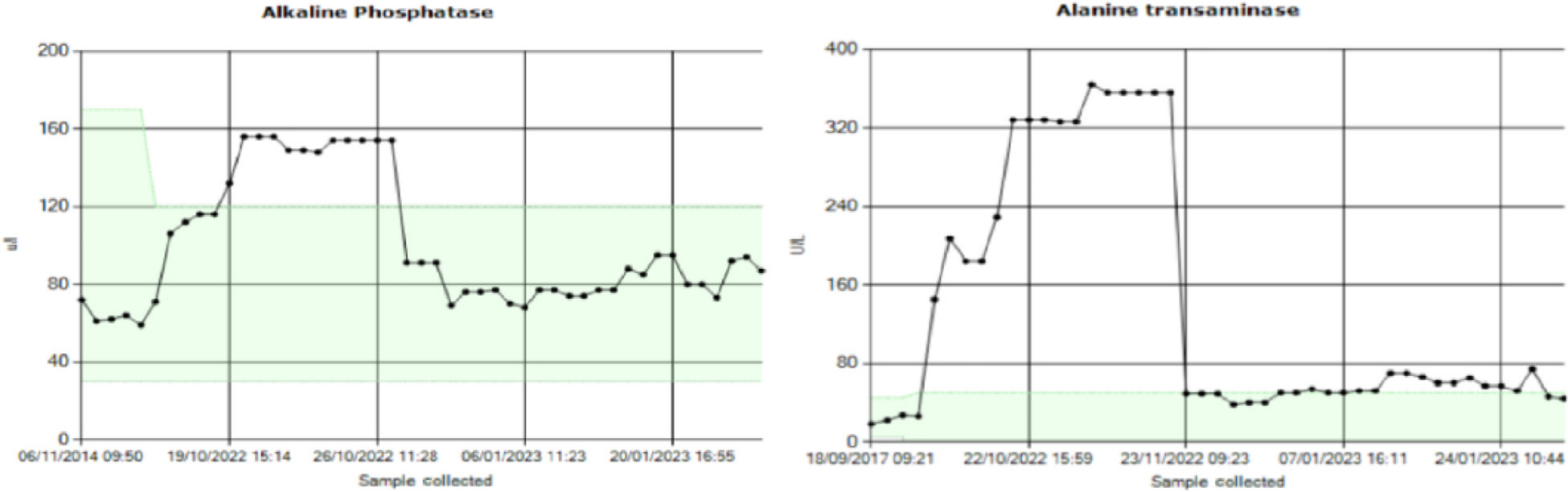
Trend of liver function tests pre and post initiation of treatment.

High dose glucocorticoids and Methotrexate were started, with rapid clinical and serological improvement. Both inflammatory markers and liver function tests had normalised one month later Fig. 1, Fig. 2 and 2.

CT-PET image demonstrating extensive vasculitis of the aorta, carotid (A), vertebral (B) and (C) subclavian.

## 2 Discussion

Giant Cell Arteritis (GCA) and Takayasu Arteritis (TA) come under the overarching category of Large Vessel Vasculitis. Systemic symptoms such as fever, malaise and weight loss are common to all these conditions.^1^ The diagnostic challenge occurs when patients present with non-specific constitutional symptoms alone.

Liver involvement is a rare but recognised association in LVV. Few studies have described cholestatic hepatitis secondary to LVV. It is reported that an increase of serum alkaline phosphatase is seen in 30–60% of newly diagnosed LVV patients.^2^, ^3^, ^4^ Serum bilirubin is typically unchanged.^2^ However, our case is unique in that LFT derangement was of a hepatocellular pattern.

Radiological abnormalities seen in the liver of LVV patients have also been described. MRCP performed in one patient demonstrated beading-type appearance of the intrahepatic ducts. A finding consistent with cholangitis. Upon the initiation of treatment repeat MRCP revealed no evidence of ductal abnormality.^5^ Other Images of the liver have also revealed granulomatous hepatitis^6^ and cavernous hemangiomas.^7^

The histological changes observed in the liver of patients with LVV are also variable. Although usually normal there have been reported cases of hepatocyte necrosis with portal and lobular inflammation.^2^

Early complications of GCA include ischaemic optic neuropathy, which can be irreversible.^1^ Patients with GCA also have a twofold increased lifetime risk of Aortic aneurysm.^2^ Therefore, early initiation of glucocorticoids is crucial.

## 3 Conclusion

There is a need to highlight the association between LVV and transaminitis. Raising awareness enables diagnosis and in turn prompt treatment.

## Patient consent

The patient in this study unfortuantely passed away. Therefore, written informed consent was obtained from his next of kin.

## Declaration of competing interest

The authors declare no conflicts of interest.
